# Survey of awareness, attitudes, and compliance with COVID-19 measures among Vermont residents

**DOI:** 10.1371/journal.pone.0265014

**Published:** 2022-03-14

**Authors:** Christine Vatovec, John Hanley

**Affiliations:** 1 Gund Institute for Environment, Larner College of Medicine, and Rubenstein School of Environment & Natural Resource, University of Vermont, Burlington, Vermont, United States of America; 2 Microbiology & Molecular Genetics, The Translational Global Infectious Diseases Research Center, Vermont Integrative Genomics Resource, Larner College of Medicine, University of Vermont, Burlington, Vermont, United States of America; Kyushu Daigaku, JAPAN

## Abstract

The objective of this research was to examine residents’ awareness, attitudes, and compliance with COVID-19 public health guidelines in Vermont, which emerged as an early leader in national pandemic response. Our methods included conducting an online survey of adult Vermont residents between January and April 2021. We analyzed demographics associated with awareness and compliance, and identified features associated with non-compliance. Our results show that of the 2,208 adult Vermont residents who completed the survey, 90% were extremely aware of the state’s COVID-19 guidelines, and 95% reported knowing exactly what to do to follow recommended actions. Political affiliation emerged as a primary factor related to attitudes and compliance. Self-identified Republicans were less likely to agree that public health measures keep people safe or help businesses stay open, and were less likely to follow masking, quarantine, social distancing, and vaccine guidance than Independents, Progressives, and Democrats. The large differences in COVID-19 infection and death rates across the country, and recent shift toward a "pandemic of the unvaccinated," underscore the need for identifying public health strategies that work in some areas in order to adapt and apply them to areas that have struggled with controlling the virus. Consistent with national surveys, our results show that resistance to public health guidance is a partisan challenge even in states with high compliance. Identifying populations that are less supportive or hesitant to follow guidelines while understanding factors that motivate compliance can help inform strategies for developing targeted programs to encourage collective action on pandemic response. Developing communication strategies that reach people who do not believe COVID-19 guidelines keep them safe is necessary to reach universal compliance.

## Introduction

Between January 2020 and January 2022, more than 57 million COVID-19 cases including over 829,000 COVID-19 deaths were reported in the United States, with high levels of variation across the country in terms of infection and death rates [1]. Public health measures including masking, social distancing, quarantine, and vaccination acceptance have all proven useful tools for responding to the pandemic [2–5]. However, wide variation in adoption and compliance with these measures has resulted in regional variation in infection rates, with higher rates in areas not following public health guidance [6]. Therefore, there is a critical need to identify factors associated with people who do not adhere to recommended public health actions in order to develop targeted public health responses to regions where the pandemic continues to spread. In this paper, we present findings from a state-wide survey of Vermont—which emerged as an early leader in pandemic response in the U.S. by consistently reporting among the lowest rates of infection and mortality during the early pandemic—that highlight aspects associated with people who report not following COVID-19 public health guidance.

In September 2020, six months into the COVID-19 pandemic, Dr. Anthony Fauci, Director of the U.S. National Institute of Allergy and Infectious Disease and chief medical advisor to the president, called Vermont’s response to the pandemic a “model for the country” [7]. Fauci praised Vermont residents for following the state’s public health guidance for mask-wearing, social distancing, avoiding crowds, and other measures that lowered the risk of SARS-CoV-2 transmission [8]. At that time, Vermont had both the lowest rate of new COVID-19 infections and test positivity in the country [1], a trend that continued through late summer 2021. On June 14, 2021, Vermont was the first state in the U.S. to reach an 80% vaccination threshold among eligible residents aged 12 years and older, and Vermont’s Republican Governor Phil Scott lifted all remaining pandemic restrictions at that time [9].

Public officials have pointed to several reasons for Vermont’s early success at curtailing the COVID-19 pandemic. Vermont’s state Health Commissioner Dr. Mark Levine identified the state’s relatively healthy population, early action to perform contact tracing and testing among the state’s most vulnerable populations, fast action to promote social distancing, consistent messaging and a coordinated approach from the state government as among the reasons for the state’s successful handling of the early pandemic [7]. Governor Scott touted following the data and trusting science for the state’s success [9]. Both Governor Scott and Commissioner Levine heralded Vermont’s residents as the key factor in allowing Vermont to emerging as an early leader in the public health response to the pandemic [7, 10]. And yet these early successes yielded as the Delta and Omicron variants of SARS-CoV-2 created what Governor Scott referred to as a “pandemic of the unvaccinated” [11], leading to the highest test positivity rate and case count in Vermont since the beginning of the pandemic.

Recent behavioral science research related to the pandemic provides a framework for understanding factors that influence people’s decisions regarding public health recommendations. In relation to following masking guidelines the cost of masks, understanding of infection risk, and social conformity all play a role in pandemic-related prosocial behavior [12]. The social dilemma of the trade-off between perceived short-term personal costs versus long-term societal benefits that result from following public health guidance can present a barrier to prosocial behaviors [13]. Importantly, social norms of mask-wearing, social distancing, and other prosocial behaviors within close peer groups can help drive compliance with recommended actions [14, 15]. In addition, policies that support social distancing have been identified as a strategy to help minimize infection rates among unvaccinated populations [16].

This article provides insight into behaviors among Vermont’s residents in response to the state’s COVID-19 guidelines, and highlights populations that report non-compliance with masking, social distancing, vaccination, and other recommended public health actions. Our hope is that this information will be useful for responding to the continuing challenges of COVID-19, along with future public health challenges across the country.

## Materials and methods

This research project and all related procedures were approved by the University of Vermont Institutional Review Board (CHRBSS 1363), and was open January 13 through April 7, 2021. Data were collected using an electronic questionnaire through Qualtrics® (Provo, UT). The landing page of the online survey instrument provided research information and a consent statement; participants who chose to continue with the anonymous survey acknowledged their consent by clicking a consent agreement button in order to continue to the survey instrument.

We obtained a convenience sample of Vermont residents by promoting the survey invitation on state agency websites (Department of Health, Department of Tourism & Marketing, Agency of Commerce and Community Development), neighborhood-based listservs, and college and university listservs (Norwich University, St. Michael’s College, University of Vermont).

### Measures

We used 5-point Likert scales to measure awareness (extremely aware to not at all aware) of Vermont’s COVID-19 public health guidelines, and agreement (strongly agree to strongly disagree) with statements regarding respondents’ perceptions of and response to the state’s guidelines (e.g., I take the recommendations from Vermont’s government authorities to prevent Covid-19 very seriously). We used a 5-point Likert scale to measure compliance (always to never) with recommended actions (e.g., To what extent do you follow each of these recommended actions: Wear a mask in public). We asked respondents to rank the sources of information where they had learned the most about Vermont’s COVID-19 guidelines (number one rank corresponded to the most used source). We also collected standard demographic data (e.g., age, gender, income, political affiliation), as well as whether the respondent had been personally affected by COVID-19 (e.g., frontline worker, lost a job, lost a loved one) and whether they had ever tested positive for COVID-19.

### Survey questionnaire and data

Survey questionnaire and the complete survey dataset are available in the Harvard Dataverse https://doi.org/10.7910/DVN/BFTPZE.

### Statistical analyses

Descriptive statistics were analyzed in Qualtrics. For all three of Vermont’s major political parties (Democrats, Progressives, Republicans) and self-described Independents, we display the percent of respondents who reported that they “Always” took an action, and the percent who reported agreement (“Agree” or “Strongly Agree”) with COVID-19 awareness questions and we present these analyses as dumbbell plots.

We employed the Conjunctive Clause Evolutionary Algorithm (CCEA) to better understand factors associated with respondents who 1) never quarantine, 2) do not take guidelines seriously, or 3) do not agree that guidelines keep them safe [17]. For each of the three outputs, we ran five seeded iterations of the CCEA and only the most-fit conjunctive clauses were selected for each output. We then extracted features from the most-fit conjunctive clauses for display in a Venn diagram to highlight which features and their associated values were shared and those that were unique to each of the three outputs.

## Results

### Characteristics of survey respondents

A total of 2,208 Vermont residents aged 18 years or older completed the survey, which represents 0.43% of the state’s adult population (Tables 1 and S1). Respondents were predominately white/Caucasian (94%), non-Hispanic (97%), female (75%), educated (69% with Bachelor’s degree or higher), and with annual income greater than $75,000 (53%). The survey sample was representative of the state’s population with regard to race and ethnicity. The survey sample included four primary political affiliations (Democrat 48%, Independent 21%, Progressive 8%, and Republican 7%), highlighting the state’s political diversity. The majority of respondents (60%) reported having been personally affected by COVID-19 in some way, and 3% had tested positive for the virus.

**Table 1:** Characteristics of respondents to survey on Vermont’s COVID-19 Guidelines among state residents aged 18 years or older between January 13 –April 7, 2021 (n = 2,208).

Variable	Vermont residents n (%)	Vermont population^1^ (%)
Gender		
Female	1513 (77)	(51)
Male	426 (22)	(49)
Non-binary	25 (1)	na
Race		
White or Caucasian	1897 (95)	(94)
Black or African American	13 (1)	(1)
American Indian or Alaska Native	21 (1)	(<1)
Asian, Indian, or Pacific Islander	27 (1)	(2)
Multiracial	33 (2)	n/a
Ethnicity		
Hispanic or Latino/a	44 (2)	(2)
Not Hispanic or Latino/a	1819 (98)	(98)
Age (years)		
18–24	140 (7)	na^2^
25–34	262 (13)	(19)
35–44	359 (18)	(11)
45–54	359 (18)	(14)
55–64	426 (21)	(16)
65–74	369 (18)	(11)
75 +	82 (4)	(7)
Income		
<$25,000	147 (8)	(21)
$25 to $49,999	362 (19)	(22)
$50 to $74,999	438 (23)	(19)
$75 to $99,999	352 (18)	(14)
>$100,000	607 (32)	(24)
Education		
Less than high school	1 (<1)	(8)
High school or equivalent	105 (5)	(29)
Some college or Associate’s degree	416 (21)	(27)
Bachelor’s degree or higher	715 (74)	(36)
Political Affiliation		
Democrat	909 (48)	(57)^3^
Independent	410 (21)	na
Libertarian	21 (1)	na
Progressive	154 (8)	na
Republican	141 (7)	(29)
None	275 (14)	na
Affected by COVID-19		
Front-line worker, healthcare	196 (10)	na
Front-line worker, other	285 (14)	na
Lost a loved one to COVID-19	125 (6)	na
Lost job because of COVID-19	128 (6)	na
Still have job but lost significant income	268 (13)	na
Business owner hurt by COVID-18	237 (12)	na
None of the above	816 (40)	na
Ever tested for COVID-19		
Yes	1189 (61)	na
• Positive test result	41 (3)	na
No	772 (39)	na

^1^ Data from United States Census Bureau https://data.census.gov/cedsci/; Vermont total population estimate for the year the survey was completed (2016): 624,594.

^2^ U.S. Census Data is for adults aged 20–34 years for age category.

^3^ Political party affiliation data from Pew Research Center 2014 Religious Landscape Study https://www.pewforum.org/religious-landscape-study/state/vermont/party-affiliation/.

### Awareness and attitudes toward Vermont COVID-19 guidelines

**Fig 1:**
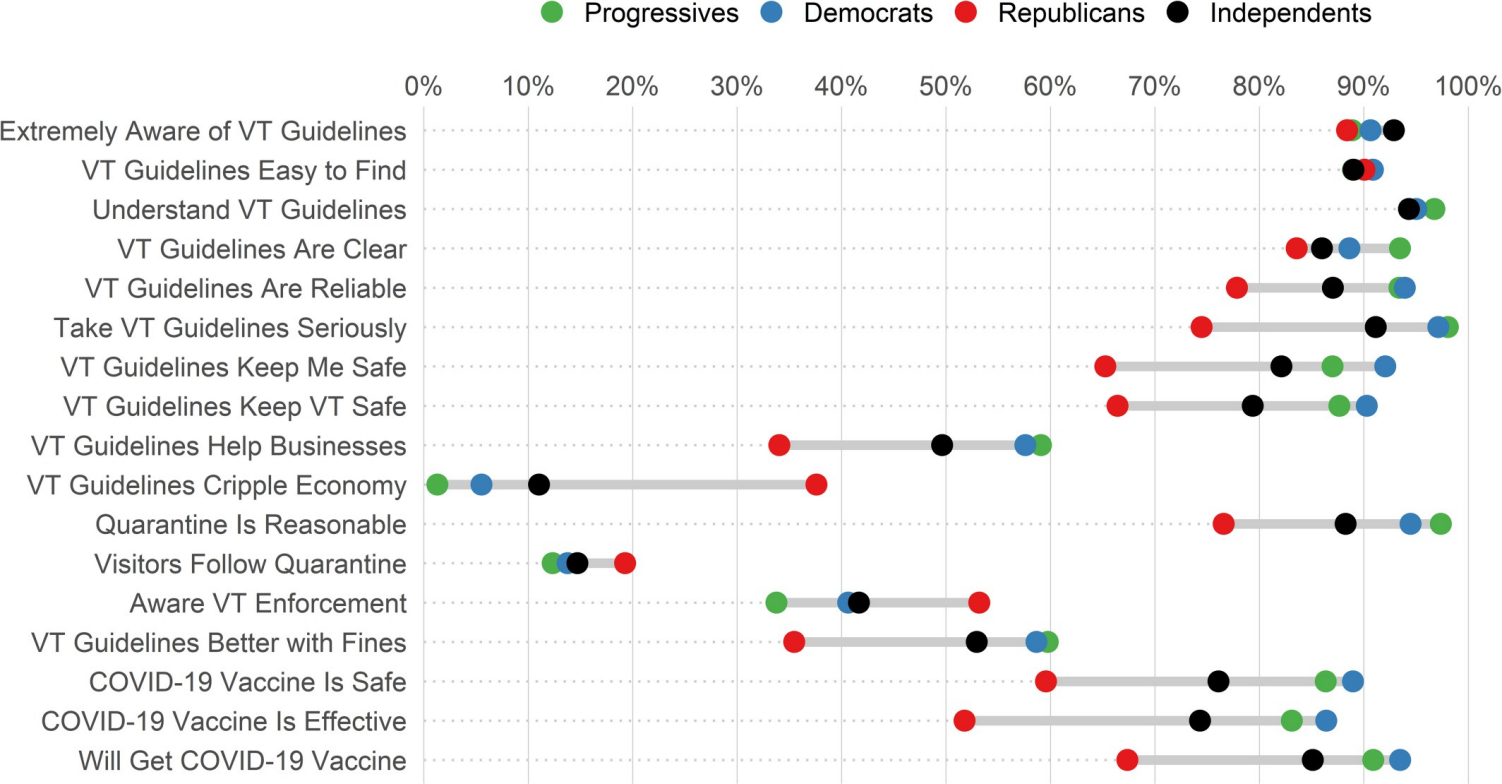
Percent of Vermont residents who responded with awareness and agreement to survey questions, delineated by self-identified political party affiliation, January–April 2021. (Note: The first line reports the percentages of respondents who reported being “Extremely Aware” of VT guidelines; all other lines report the percentage who responded “Agree” or “Strongly Agree” with survey statements).

Among all survey respondents, 89% (95% CI: 90–91%) were extremely aware of Vermont’s COVID-19 guidelines, 93% (95% CI: 94–96%) had received information from the state Department of Health, 90% (95% CI: 89–91%) agreed that the guidelines were easy to find, 92% (95% CI: 91–93%) reported taking the recommendations very seriously, and 94% (95% CI: 93–95%) said that they understood what to do to follow the guidelines (Fig 1). Political party was strongly associated with several beliefs about state guidelines. A smaller percentage of people who self-identified as Republican reported agreement with statements that Vermont’s COVID-19 guidelines are reliable, keeping me safe, keeping Vermonters safe, and helping keep businesses open, as compared to Independents, Democrats, and Progressives. Similarly, beliefs around COVID-19 vaccine safety and effectiveness varied by political party affiliation with 52% (95% CI: 46–60%) of Republicans and 86% (95% CI: 84–88%) of Democrats in agreement that the vaccine is effective. Agreement that the respondent would get the vaccine as soon as it became available to them ranged from 67% (95% CI: 59–75%) among Republicans to 93% (95% CI: 91–95%) among Democrats.

We found two instances where the partisan trend was reversed: 1) 38% (95% CI: 30–46%) of Republicans agreed that Vermont’s guidelines cripple the economy compared to 6% (95% CI: 4–8%) of Democrats and 1% (95% CI: 0–3%) of Progressives, and 2) 52% (95% CI: 44–60%) of Republicans reported awareness of Vermont’s enforcement of COVID-19 guidelines compared to 41% (95% CI: 38–44%) of Democrats and 33% (95% CI: 26–39%) of Progressives. Interestingly, less than 20% of respondents from all four political parties agreed that visitors to the state follow quarantine guidelines.

### Behaviors related to Vermont COVID-19 guidelines

**Fig 2:**
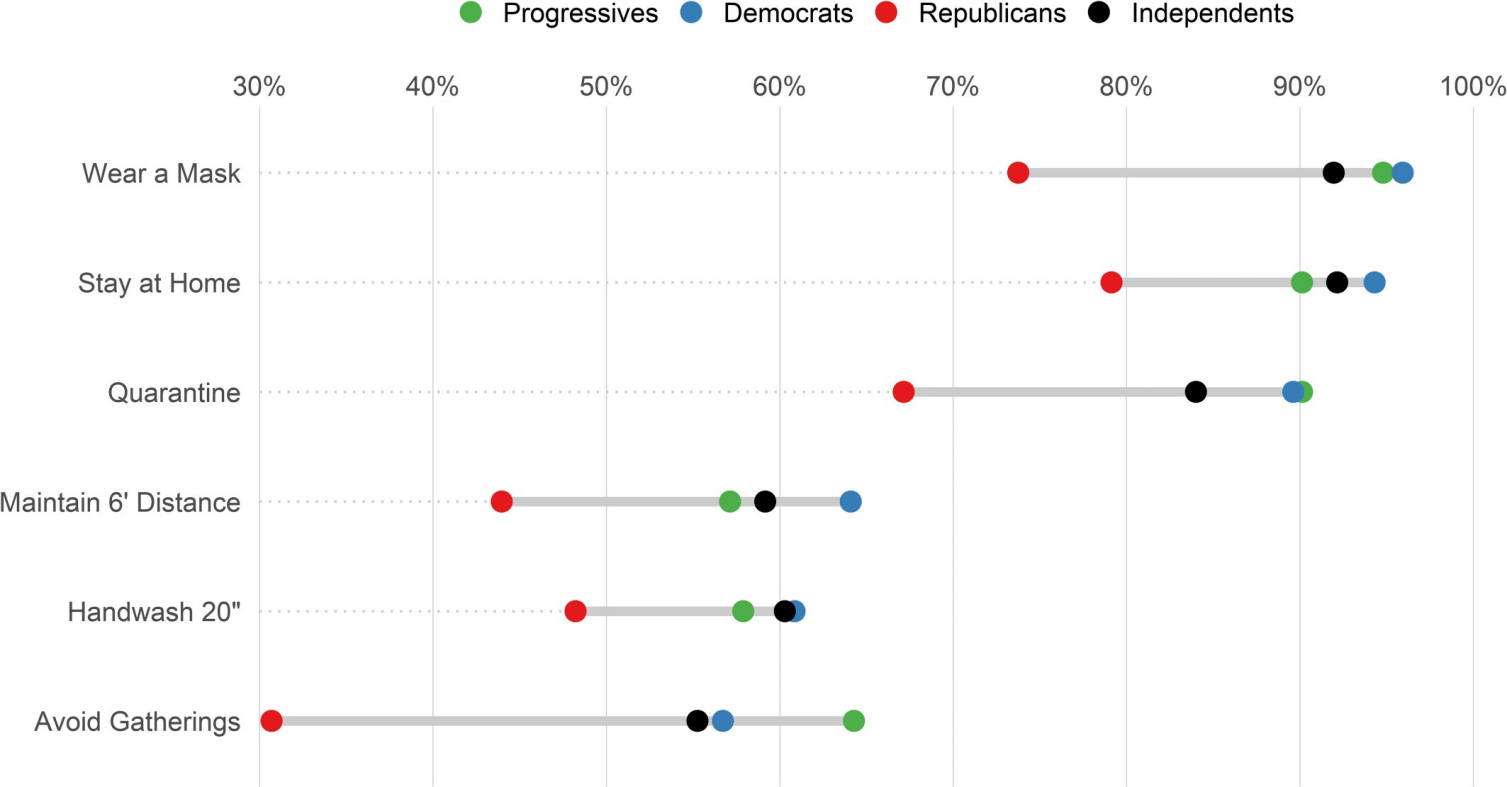
Percent of Vermont respondents who reported always following recommended actions to prevent COVID-19 infection, delineated by self-identified political party affiliations, January–April 2021.

Compliance with Vermont’s COVID-19 public health guidance also appear to follow partisan lines. Republicans consistently had the lowest percentage of respondents who always followed recommended actions to prevent COVID-19 transmission (Fig 2). For example, more Progressives (95%; 95% CI: 92–98%), Democrats (96%; 95% CI: 95–97%), and Independents (92%; 95% CI: 89–95%) reported always wearing a mask compared to 73% (95% CI: 66–80%) of Republicans. Always avoiding social gatherings had the largest difference in response rates, ranging from 31% (95% CI: 23–39%) of Republicans to 55% (95% CI: 50–60%) of Independents, 57% (95% CI: 54–60%) of Democrats, and 64% (95% CI: 56–72%) of Progressives.

### Factors associated with responses to Vermont’s public health guidance

News sources where Vermonters received information about Vermont’s COVID-19 guidelines also varied by political affiliation. Online news was the top ranked source of information on Vermont’s COVID-19 guidelines among Progressives (45%; 95% CI: 37–53%), Democrats (42%; 95% CI: 39–45%), and Independents (41%; 95% CI: 36–46%), and the second ranked source among Republicans (32%; 95% CI: 26–40%). Television was the number one news source among Republicans (35%; 95% CI: 27–43%; S1 Fig).

Among all respondents, the primary motivating factors for following the state’s pandemic-related guidelines were “my own health” (41%; 95% CI: 39–43%), “the health of my friends/family” (40%; 95% CI: 38–42%), and “the health of other Vermonters” (17%; 95% CI: 15–19%; S2 Fig). Political affiliation was not associated with a significant difference in motivation for following guidelines, but did suggest some trends in altruism among Progressive of whom a greater percentage ranked health of family as their primary motivation, as compared to Democrats, Independents, and Republicans who had a higher percentage of respondents who gave the top rank to their own personal health (S3 Fig).

**Fig 3:**
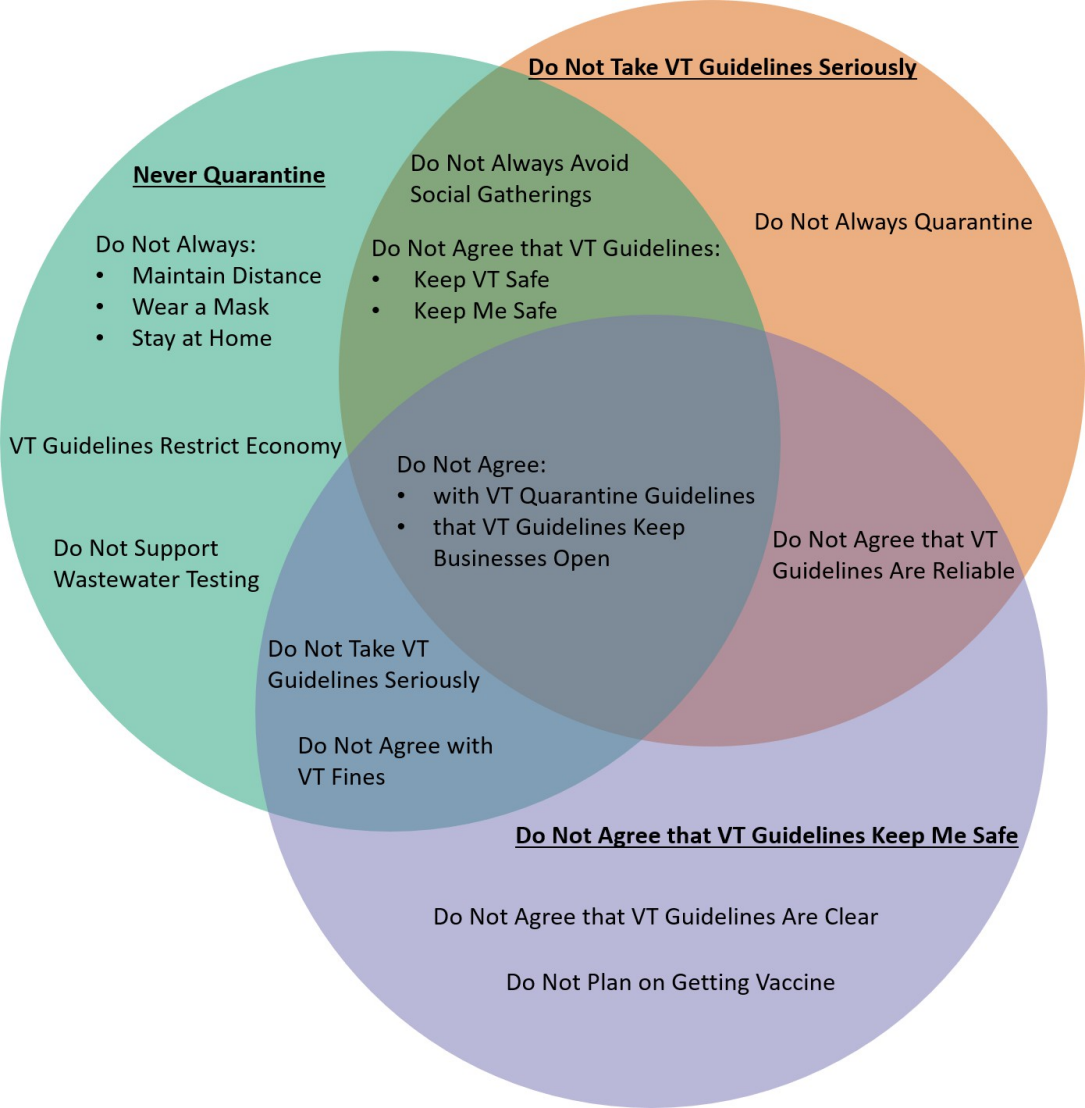
Factors associated with respondents who responded negatively to Vermont’s COVID-19 guidelines, January–April 2021.

While the majority of survey participants responded positively to questions about Vermont’s COVID-19 guidelines (Figs 1 and 2), we wanted to explore the features associated with the minority of people who reported that they 1) never quarantined, 2) did not take the guidelines seriously, and 3) did not think the guidelines kept them safe. Fig 3 shows the overlap between respondents in these groups; the center of the diagram highlights that the associations between all three of these minority groups were that they did not agree with Vermont’s guidelines and did not agree that the guidelines keep businesses open. We also found overlap between these three minority groups. For instance, respondents who do not take the guidelines seriously are also associated with never quarantining and do not agree that the guidelines keep them safe. In addition, while the overwhelming majority of Vermont residents agreed that the state guidelines were clear, there is an association between those who did not find guidelines to be clear and those who do not agree that the guidelines keep them safe.

## Discussion

Our study examined Vermont residents’ beliefs and response to the state’s COVID-19 public health recommendations between January and April 2021 in an effort to provide some insight into the state’s relative early success in responding to the pandemic. During that time period, state officials held twice-weekly press briefings to provide updates on virus transmission and public health measures, including a phased roll-out of the COVID-19 vaccine for residents. Our results show that awareness and understanding of the state’s pandemic response plan were high, as was the overall percentage of respondents who agreed that pandemic-related public health communications was both clear and reliable. These survey outcomes support the idea that a clear, consistent, and coordinated public health campaign is likely to result in a higher rate of compliance with public health recommendations and mandates [18–20].

During our survey period, Vermont reported fewer than 20 new cases per 100,000 residents each day, and the governor’s (Republican) management of the pandemic had a 71% approval rating. Nationally, the proportion of Americans who approved of their local elected officials varied by political partisanship with 41% of Republicans approving, compared with 57% of Democrats [21], a trend that was intensified in our results (55% Republican approval, 85% Democratic) [22]. Also consistent with national trends [21, 23], our findings indicate that political affiliation was a major factor in Vermonters’ attitudes toward and compliance with the state’s COVID-19 public health guidelines. Respondents who identified as Republican reported the lowest rates of compliance with all COVID-19 guidelines including masking, quarantine, social distancing, and vaccination as compared with all other major political parties in the state of Vermont. Observed national trends in partisan-driven non-compliance with physical distancing has been associated with higher rates of COVID-19 infection and mortality [24], and is an area that may be worth pursuing for future investigation in Vermont.

Since vaccination against COVID-19 is both a recommended public health action and a major determinant for many state governments to relax restrictions [25], it is important to note that our study shows that Vermonters’ intention to be vaccinated (86%) was slightly higher than the national average (75%), and again differed by political affiliation (67% Republicans, 96% Democrats) [26]. The partisan gap in relation to Vermont’s pandemic response may be partially explained by our findings that Republicans were more likely to think guidelines would cripple the economy and more pessimistic on whether the guidelines would help Vermont businesses to remain open. Nationally, consumption of conservative media such as Fox News has been associated with reduced physical distancing and higher COVID-19 infection and mortality rates [24] and our findings that television was the primary news source among Republicans may suggest another potential driver of the partisan response to Vermont’s pandemic guidelines.

Despite political partisanship in attitudes and compliance, the majority of all respondents ranked their primary motivations for following recommended public health actions as protecting their own health, the health of family and friends, and the health of other Vermonters. This finding is consistent with a large international survey [27], and supports the idea that public health messaging targeting these motivating factors may be one component of strategies that can cut across political divides.

While our results show that Vermont residents have high levels of awareness regarding COVID-19 guidelines and adherence to actions to limit transmission, the emergence of the COVID-19 Delta variant showed that those who do not take the guidelines seriously or adhere to actions that limit the transmission of COVID-19 are likely to be the most vulnerable to infection [11, 28]. Therefore, it is critical to increase understanding of those features that best describe people who do not take seriously or comply with recommended COVID-19 public health measures. Our analysis indicates a number of features that help describe these populations (Fig 3). Respondents who do not agree with Vermont’s guidelines, do not agree that the guidelines keep business open, do not agree that the guidelines keep them safe, and do not take the guidelines seriously are common features across all three questions we examined. Thus, for a minority of Vermont residents there is perhaps a communication issue or possibly a philosophical disagreement with Vermont’s guidelines. The most concerning association is between respondents who do not believe that the guidelines keep them safe and do not agree that the guidelines are clear. Unfortunately, there are no strong correlations in our demographic data that could help the state of Vermont better target this population. It would also be helpful to understand why this group does not feel that Vermont’s guidelines are clear and how the messaging could be improved so that this group can fully understand the guidelines and hopefully adhere to them.

Respondents who do not feel that the guidelines keep them safe are also uniquely associated with the response that they do not plan to receive the vaccine. Although Vermont currently leads the nation in vaccinations [9], the state has not achieved universal full vaccination among eligible residents despite an abundance in vaccine availability. Again, this association between respondents not planning on getting vaccinated and not believing that the guidelines keep them safe could relate to a communication issue, suggesting that more work is needed on communicating the science behind non-pharmaceutical interventions and vaccination in limiting COVID-19 transmission and deaths [20, 29, 30].

In terms of actions, perhaps it is not surprising that those who never quarantine are also not likely to always adhere to other actions such as masking, maintaining physical distance, staying at home, and avoiding gatherings. Such lack of enthusiasm to limit COVID-19 transmission through non-pharmaceutical interventions makes this group particularly vulnerable to infection. A further understanding of whether lack of consistent adherence to actions is driven by external factors such as the nature of employment or not believing in the science behind non-pharmaceutical interventions is needed to reach universal compliance.

### Limitations

While our study offers insights on pandemic response from a state that has had overall positive outcomes, several limitations are worth noting. First, since the survey sample under-represented males, low income earners, Republicans, and Vermonters with lower educational attainment as compared with Vermont’s general population, our results may not fully represent the beliefs and behaviors of these groups. However, the sample does include a representative sample of racially and ethnically diverse Vermonters which is important because of the historical lack of representation of these communities. Second, the survey tool was only provided online and in English, thereby limiting the sample to English-speaking Vermonters with internet access. Finally, the study relied on self-reporting and may reflect under- or over-reporting of Vermonters’ beliefs and behaviors. Despite these limitations, data regarding community response to pandemic-related guidelines is a necessary step toward future public health communications.

## Conclusion

The large differences in COVID-19 infection and death rates across the country underscore the need for identifying public health strategies that work in some areas in order to adapt and apply them to areas that have struggled with controlling the virus. Understanding differences in attitudes and compliance with public health measures such as masking, social distancing, and vaccination are critical for enhancing current and developing future pandemic response guidelines and communications. Identifying populations that are less supportive or hesitant to follow guidelines while understanding factors that motivate compliance can help inform strategies for developing targeted programs to encourage collective action on pandemic response.

## Supporting information

S1 Table Additional characteristics of respondents to survey on Vermont’s Covid-19 guidelines among state residents aged 18 years or older between January 13 –April 7, 2021 (n = 2,208). (DOCX)

S1 Fig News sources where respondents have received information on Vermont’s COVID-19 Guidelines, ranked from most used (1) to least used (7); numbers within cells represent percent of respondents (Note: Each rank could be applied to multiple sources). (TIF)

S2 Fig Motivation for following Vermont’s COVID-19 guidelines, ranked from most (1) to least (6) motivating factors (Note: Numbers within rankings represent sample size). (TIF)

S3 Fig Motivation for following Vermont’s COVID-19 guidelines, ranked from most (1) to least (6) motivating factors (Note: Numbers within rankings represent sample size). (TIF)
