# Supplementary material for: Survey of awareness, attitudes, and compliance with COVID-19 measures among Vermont residents
Source: PLoS One. 2022 Mar 14;17(3):e0265014. doi: 10.1371/journal.pone.0265014 (PMC8920266; doi:10.1371/journal.pone.0265014)
Supplement: S1 Table — (DOCX) [file pone.0265014.s001.docx]

**Table S1.** Additional characteristics of respondents to survey on Vermont’s Covid-19 Guidelines among state residents aged 18 years or older between January 13 – April 7, 2021 (n = 2,208).

| Variable | **Vermont residents**  **n (%)** | **Vermont population ^1^**  **(%)** |
| --- | --- | --- |
| Occupational Status |  |  |
| Part-time student | 41 (2) | na |
| Full-time student | 149 (7) | na |
| Part-time employed | 272 (13) | na |
| Full-time employed | 1055 (50) | na |
| Unemployed | 105 (5) | (2.9)^2^ |
| Retired | 371 (17) | na |
| Other | 137 (6) | na |
| Health insurance |  |  |
| Private insurance | 1305 (69) | (55) |
| Medicare/Medicaid | 547 (29) | (39) |
| Uninsured | 37 (2) | (4) |
| Vermont County of Residence |  |  |
| Addison | 51 (3) | (6)^3^ |
| Bennington | 93 (5) | (6) |
| Caledonia | 39 (2) | (5) |
| Chittenden | 856 (43) | (26) |
| Essex | 8 (<1) | (1) |
| Franklin | 58 (3) | (8) |
| Grand Isle | 7 (<1) | (1) |
| Lamoille | 82 (4) | (4) |
| Orange | 31 (2) | (5) |
| Orleans | 26 (1) | (4) |
| Rutland | 385 (19) | (9) |
| Washington | 176 (9) | (9) |
| Windham | 66 (3) | (7) |
| Windsor | 110 (6) | (9) |

^1^ Data from United States Census Bureau <https://data.census.gov/cedsci/>; Vermont total population estimate for the year the survey was completed (2016): 624,594.

^2^ Vermont unemployment rate as of April 2021 <https://labor.vermont.gov/press-release/vt-unemployment-jobs-report-april-2021>

^3^ Vermont population by county data as of October 2020 available at <https://www.healthvermont.gov/health-statistics-vital-records/vital-records-population-data/vermont-population-estimates>
