# Supplementary figures and images for: Survey of awareness, attitudes, and compliance with COVID-19 measures among Vermont residents
Source: PLoS One. 2022 Mar 14;17(3):e0265014. doi: 10.1371/journal.pone.0265014 (PMC8920266; doi:10.1371/journal.pone.0265014)

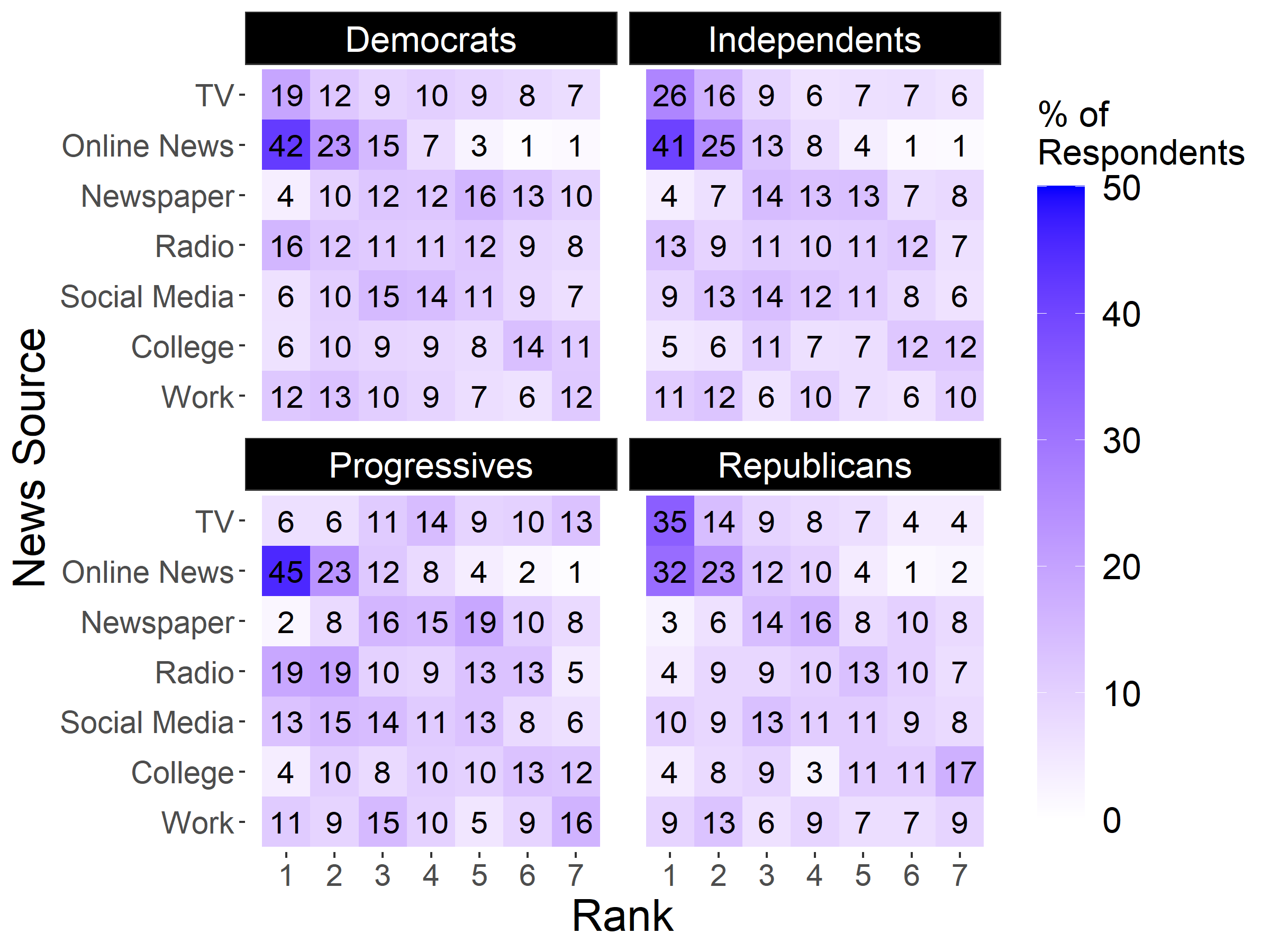

Supplement: S1 Fig — (TIF) [file pone.0265014.s002.tif]

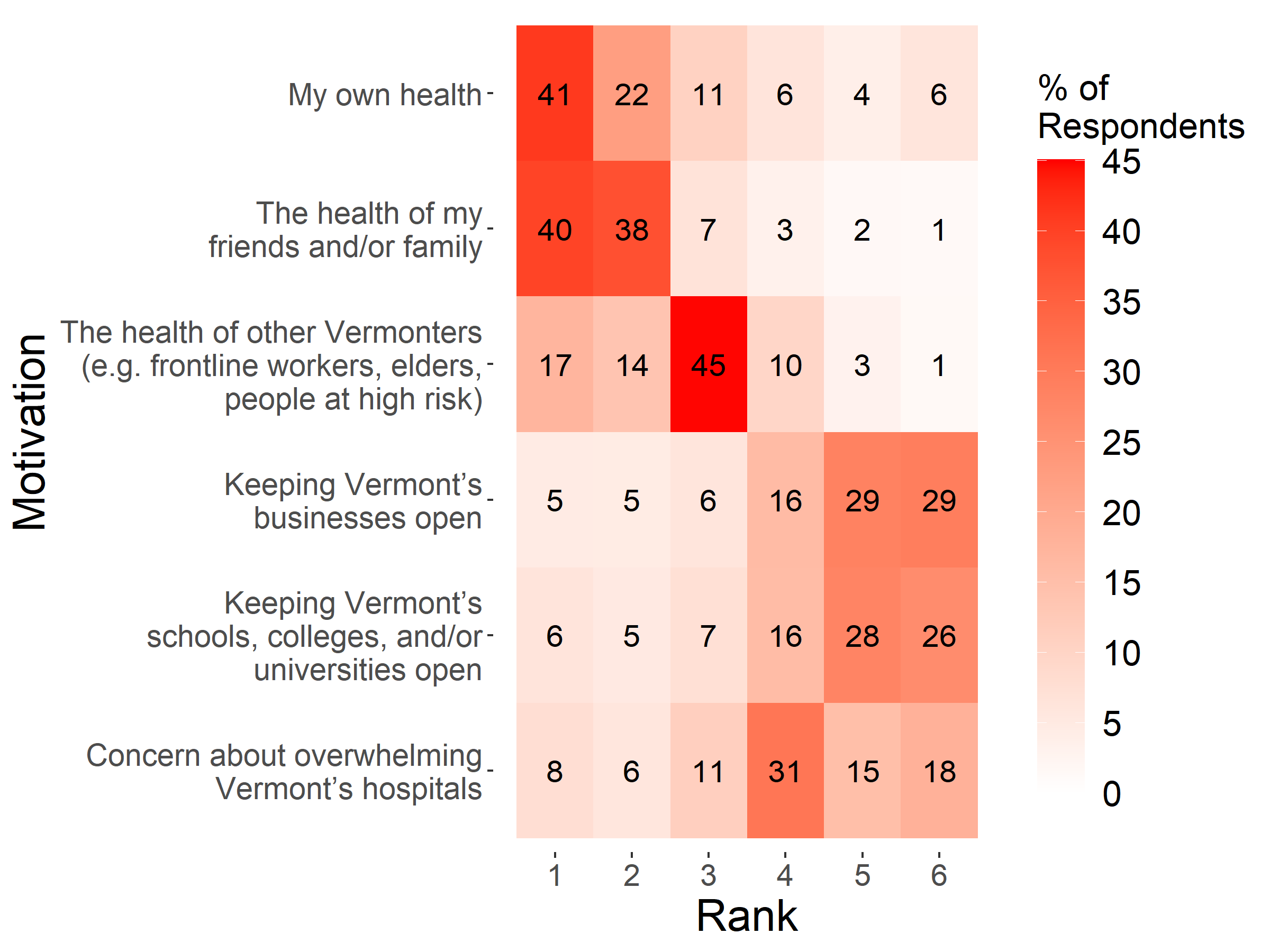

Supplement: S2 Fig — (TIF) [file pone.0265014.s003.tif]

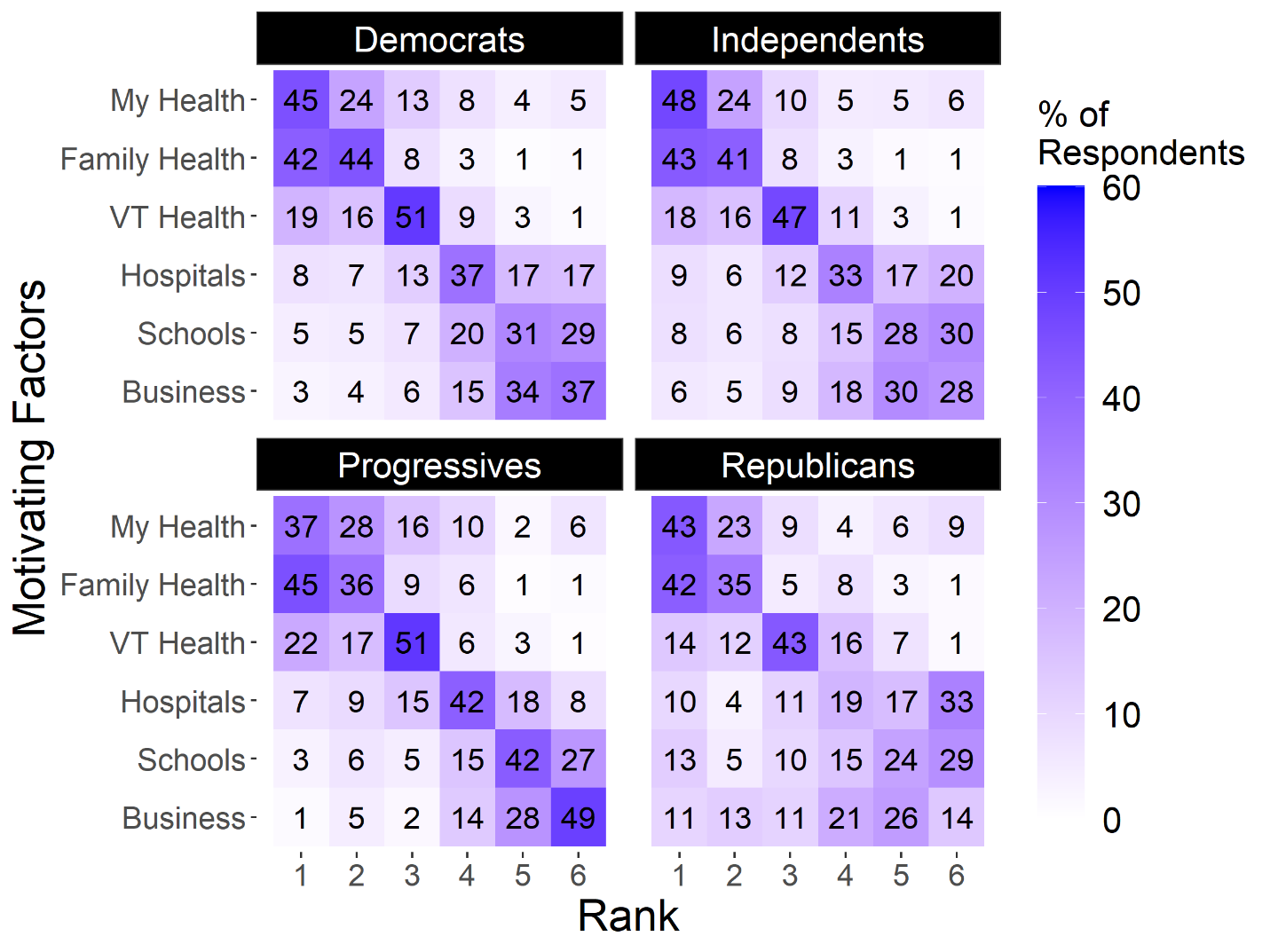

Supplement: S3 Fig — (TIF) [file pone.0265014.s004.tif]
